# Acromegaly and thyroid cancer: analysis of evolution in a series of patients

**DOI:** 10.1186/s40842-020-00113-4

**Published:** 2020-11-17

**Authors:** Karina Danilowicz, Soledad Sosa, Mariana Soledad Gonzalez Pernas, Elizabeth Bamberger, Sabrina Mara Diez, Patricia Fainstein-Day, Alejandra Furioso, Mariela Glerean, Mirtha Guitelman, Débora Katz, Nicole Lemaitre, Alicia Lowenstein, Mariela del Valle Luna, María Paz Martínez, Karina Miragaya, Daniel Moncet, María Victoria Ortuño, Analía Pignatta, Constanza Fernanda Ramacciotti, Adriana Reyes, Amelia Susana Rogozinski, Patricia Slavinsky, Julieta Tkatch, Fabián Pitoia

**Affiliations:** 1 Endocrinology Division, Hospital de Clínicas José de San Martín- Universidad de Buenos Aires, Buenos Aires, Argentina; 2 Neuroendocrine Department, Sociedad Argentina de Endocrinología y Metabolismo, Buenos Aires, Argentina; 3 Endocrinology Division, FLENI, Buenos Aires, Argentina; 4 Centro Privado de Endocrinología, Mendoza, Argentina; 5 Endocrinology Division, Hospital Pirovano, Buenos Aires, Argentina; 6 Department of Endocrinology and Nuclear Medicine, Hospital Italiano de Buenos Aires, Buenos Aires, Argentina; 7 Endocrinology Division, Hospital Ramos Mejía, Buenos Aires, Argentina; 8 Endocrinology Division, Hospital Carlos G. Durand, Buenos Aires, Argentina; 9 Endocrinology Division, Hospital Ángel C. Padilla, Tucumán, Argentina; 10 Endocrinology Division, Hospital Alemán, Buenos Aires, Argentina; 11 Endocrinology Division, Sanatorio Güemes, Buenos Aires, Argentina; 12 Endocrinology Division, Hospital Privado de Comunidad, Mar del Plata, Buenos Aires, Argentina; 13 Endocrinology Division, Hospital Interzonal San Juan Bautista, San Fernando del Valle de Catamarca, Catamarca, Argentina; 14 Endocrinology Division, Hospital Privado Universitario de Córdoba, Córdoba, Argentina

**Keywords:** Acromegaly, Thyroid, Neoplasms, Thyroid nodule, Thyroid cancer

## Abstract

**Background:**

Acromegaly is associated with higher morbidity and mortality mainly due to cardiovascular disease. Data on the incidence and evolution of thyroid cancer in acromegaly are controversial. Our objective was to describe the characteristics of a group of acromegalic patients with differentiated thyroid carcinoma (DTC) and analyze their evolution.

**Methods:**

This is a retrospective multicenter study of 24 acromegalic patients with DTC. The AJCC Staging System 8th Edition was used for TNM staging, and the initial risk of recurrence (RR), initial response and response at the end of follow-up (RFU) were defined according to the 2015 ATA Guidelines. As a control group, 92 patients with DTC without acromegaly were randomly included. Statistical analyses were done using SPSS Statistics 20.0.

**Results:**

Median age of patients at diagnosis of acromegaly was 49.5 years (range 12–69). The median delay in diagnosis of acromegaly was 3 years (range 0.5–23). Mean baseline IGF-1 level was 2.9 ± 1.1 ULN. Median age at DTC diagnosis was 51.5 years (18–69).

At the moment of diagnosis of DTC, 58.3% of the patients had active acromegaly. Median time from DTC diagnosis to acromegaly control was 1.25 years (0.5–7). Mean DTC tumor diameter of the biggest lesion was 14.6 ± 9.2 mm, being multifocal in 37.5%. All tumors were papillary carcinomas, two cases being of an aggressive variety. Lymph node dissection was performed in 8 out of 24 patients and 62.5% had metastases. Only one patient had distant metastases. Radioiodine ablation was given to 87.5% of patients. Nineteen patients (79%) were stage I, four (17%) stage II and one (4%) stage IVb.

Initial RR was low in 87% (21/24), intermediate in 9% (2/24) and high in 4% (1/24) patient. RFU was: 83% (19/23) patients with no evidence of disease, 9% (2/23) with indeterminate response, 4% (1/23) with biochemical incomplete response and 4% (1/23) with structural incomplete response, at a median time of FU of 36.5 months.

When comparing RFU between acromegalics and controls no statistically significant differences were found.

**Conclusions:**

Patients with acromegaly and DTC mostly had a low initial RR. When compared with the control group, we found that DTC patients with acromegaly did not have a worse evolution.

**Supplementary Information:**

The online version contains supplementary material available at 10.1186/s40842-020-00113-4.

## Background

Acromegaly is considered a rare chronic debilitating disease, mainly caused by a somatotroph adenoma in more than 95% of cases [1]. It is associated with excessive levels of GH and IGF-1, with somatic overgrowth and physical disfigurement. Higher morbidity and mortality, mainly due to cardiovascular and cerebrovascular disease, are described [2].

The prevalence of acromegaly ranges between 2.8 to 14 cases per 100,000 inhabitants [3, 4]. Recent studies show a standardized mortality ratio (SMR) of 1.16 to 2.14 [5, 6], lower than decades before, as a result of the improvement in diagnosis and therapy. At follow-up, a serum GH level lower than 1 ng/ml is considered a biochemical objective, and it has been related to an increase in survival [7], being GH levels with cumulative GH exposure the best predictors of a decrease in mortality [8].

Long-term exposure to GH, with the concomitant increase in IGF-1, has proliferative and antiapoptotic effects, which might explain the higher frequency of neoplasms in this group of patients [9–11]. However, data on the incidence of cancer in acromegaly are inconclusive [12, 13].

In the German Acromegaly Registry, no evidence of higher overall incidence of cancer was found [14]. Dal et al. examined whether patients with acromegaly were at higher risk of cancer in a nationwide cohort study (from 1978 to 2010) including 529 acromegaly cases. Incident cancer diagnosis and mortality were compared with national rates estimating standardized incidence ratios (SIRs). Cancer incidence rates were slightly elevated in patients with acromegaly [15]. A retrospective multicenter epidemiological study of a total of 1512 Italian acromegalic patients found that mortality was significantly higher in patients with persistently active disease (1.93; 95% confidence interval -CI-:1.34–2.70), the main causes of death being vascular diseases and malignancies with similar prevalence. The multivariate analysis showed that older age, higher GH at last follow-up, higher IGF-1 levels at diagnosis, malignancy, and radiotherapy were independent predictors of mortality [16].

Some publications have shown an increased prevalence of multinodular goiter in patients with acromegaly as well as an increased risk of thyroid cancer, 3.2 versus 0.3% compared with controls [17]. The pathophysiology is related to the proliferative and antiapoptotic effect of IGF-1 on thyroid cells.

The objective of this study was to describe clinical and biochemical characteristics in a group of acromegalic patients with differentiated thyroid carcinoma (DTC) and to identify any predicting factors for DTC evolution. Another objective was to analyze recurrence risk (RR) and response at the end of follow-up (RFU), comparing the outcomes with non-acromegalic patients with DTC (control group).

## Methods

This was a retrospective multicenter study that included 24 patients with acromegaly and DTC. Acromegaly control or remission was defined as IGF-1 ≤ 1 upper limit of normality (ULN) with or without medical treatment (MT) respectively. The AJCC Staging System, 8th Edition was used for TNM staging, and the initial RR, initial response and RFU were defined according to the American Thyroid Association Guidelines 2015 [18]. A descriptive analysis of the clinical characteristics of the patients was carried out.

A comparative analysis was performed between acromegalic patients with available RFU and control patients with DTC without acromegaly (1:4 ratio). Control patients according to RR were automatically randomly selected from a database of DTC patients treated in one of the centers involved in the study. These patients had the same regional origin as those treated in the other centers.

The statistical analysis was carried out using the SPSS Statistics program (20.0.version). Results were expressed as mean ± DS for normally distributed variables, and median (with range) for non-normally distributed variables. IGF-1 levels were expressed according to the ULN. Frequencies are shown as percentages. A *P* < 0.05 was considered to be statistically significant. The Kruskal-Wallis or the Wilcoxon test was used to analyze differences in continuous variables between study groups where the variables were not normally distributed and the Fisher’s exact test was used to compare categorical variables between groups. For comparing RFU between groups, the Chi2 test was applied and, after recategorizing responses into excellent and non-excellent (aggregating high risk categories by including indeterminate, biochemical incomplete and structural incomplete responses) the Odds Ratio (OR) was estimated.

## Results

A total of 24 patients with acromegaly and DTC were included in the study. Eighteen (75%) were women. Median age at diagnosis of acromegaly was 49.5 years (range 12–69 years). The median delay in diagnosis of acromegaly was 3 years (range 0.5–23 years), estimated according to personal history of signs and symptoms suggestive of acromegaly. Baseline IGF-1 at diagnosis was 2.9 ± 1.1 ULN. The mean tumor diameter was 18.3 ± 12.6 mm, with empty sella in four patients.

Transsphenoidal surgery had been performed in 85% (17/20). The mean lowest postoperative IGF-1 level was 1.6 ± 1.2 ULN. Most of the patients (87.5%, 21/24) needed additional medical treatment. Median duration of medical treatment was 33.5 months (range 5–132 months), and 71.4% (15/21) of the patients achieved biochemical control. Seven patients (29%, 7/24) received adjuvant radiotherapy. Median time from the diagnosis of acromegaly to biochemical control was 22.5 months (range 6–132 months).

Personal history of other tumors was found in 16.7% (4/24, one with breast cancer, three with colon neoplasms: one rectum adenocarcinoma and two colonic adenomas with low grade dysplasia).

Median age at DTC diagnosis was 51.5 years (range 18–69 years). 46% (11/24) had palpable nodules. No patient had a personal history of cervical irradiation. Most patients (87.5%, 21/24) had normal thyroid function tests with negative thyroid antibodies in 71% (17/24) before surgery.

At the moment of diagnosis of DTC, 58.3% of the patients (14/24) had active acromegaly, with mean serum IGF-1 levels of 2.3 ± 1.1 ULN. Median time from DTC diagnosis to acromegaly control was 1.25 years (range 0.5–7 years). All patients received total thyroidectomy. Mean tumor diameter of the biggest lesion was 14.6 ± 9.2 mm, being multifocal in 37.5% (9/24). All were papillary carcinomas, 67% (16/24) classic variant and the rest follicular variant. In only two cases an aggressive variety was found (tall cell). In 33.3% (8/24) lymph node dissection was done, 62.5% (5/8) with nodal metastases. Only one patient had distant metastases (subcentimetric lung nodules). Most patients (87.5%, 21/24) received radioiodine ablation under induced hypothyroidism and the mean dose was 105 ± 58.7 mCi. Baseline characteristics were compared between the acromegalic patients and the control group: no statistically significant differences were found in gender distribution, personal history of other cancer, mean age at diagnosis of DTC, histology, tumor size, lymphadenectomy, radioablation or median time of FU. On the contrary, there was a significant difference in the frequency of insulin resistance, which was higher in acromegalics (Table 1).

**Table 1:** Characteristics and evolution of acromegalic and control patients (absolute values and percentages)

Patients	Acro (*n* = 23)	Controls (*n* = 92)	*p*-value
**Gender** [Female: n (%)]	18 (78.3)	82 (89.1)	0.29
**Mean age at DTC diagnosis** (years ± SD)	49.3 ± 13.9	47.5 ± 16.5	0.31
**Insulin resistance** [n (%)]	9 (39.1)	10 (10.9)	**0.003**
**History of other cancer** [n (%)]	2 (8.7)	3 (3.3)	0.56
**Histology**^**a**^ [n (%)]			
clPTC	16 (69.6)	73 (79.3)	0.43
fvPTC	7 (30.4)	19 (20.7)	
**Mean tumor size** [mm, (range)]	15.2 (4–35)	14.6 (3–60)	0.76
**Lymphadenectomy** [n (%)]	8 (34.8)	15 (16.3)	0.09
**Node metastases** [n (%)]	5 (62.5)	10 (66.7)	1
**Radioiodine ablation** [n (%)]	21 (91.3)	63 (68.5)	0.052
**Median iodine dose** [mCi, (range)]	100 (30–300)	100 (30–150)	0.53
**Median time of follow-up** [months, (range)]	36.5 (6–120)	49.5 (6–219)	0.14
**Follow-up response**^**b**^ [n (%)]			
**NED**	19 (82.7)	60 (65.2)	0.36
**Ind**	2 (8.7)	22 (23.9)	
**BI**	1 (4.3)	3 (3.3)	
**SI**	1 (4.3)	7 (7.6)	

^a^Histology: *clPTC* classic papillary thyroid carcinoma. *fvPTC* follicular variant papillary thyroid carcinoma

^b^Follow-up response: *NED* no evidence of disease, *Ind* indeterminate, *BI* biochemical incomplete, *SI* structural incomplete

The stratification of acromegalic patients with DTC according to the AJCC/TNM Staging System, 8th edition and the distribution of patients according to RR, initial response and RFU according to ATA 2015 are shown in Table 2.

**Table 2:** Initial DTC stage and response to treatment of acromegalic patients

**Stage**	
**I**	19/24 **(79%)**
II	4/24 (17%)
IVb	1/24 (4%)
**Initial RR**	
**Low**	21/24 **(87%)**
Intermediate	2/24 (9%)
High	1/24 (4%)
**Initial response**	
**Excellent**	12/23 (52%)
Indeterminate	7/23 (31%)
Biochemical incomplete	3/23 (13%)
Structural incomplete	1/23 (4%)
**RFU**	
**No evidence of disease**	19/23 **(83%)**
Indeterminate	2/23 (9%)
Biochemical incomplete	1/23 (4%)
Structural incomplete	1/23 (4%)

*RR* recurrence risk, *RFU* response at the end of follow-up

No statistically significant correlations were found when analyzing characteristics of acromegalic patients (age at diagnosis of DTC, time from diagnosis of DTC to control of acromegaly, IGF-1 at the diagnosis of DTC and insulin resistance) with stage, initial RR, initial response and RFU (Table 3 and Supplemental Table 1).

**Table 3:** Analysis of potential risk factors for unfavorable DTC evolution in acromegalic patients classified according to stage, initial recurrence risk, initial and follow-up response

Potential risks factors for unfavorable DTC evolution	Age at diagnosis of DTC (years) Median (range)	IGF-1 at diagnosis of DTC (ULN) Median (range)	Time between DTC diagnosis and acromegaly control (years) Median (range)	Insulin resistance n (%)
**Stage** **(*n*** **= 24)**				
**1 (*n*** **= 19)**	–	1.19 (0.32–4.8)	0.83 (0–7)	8 (42.1)
**2 (*n*** **= 4)**		1.52 (1.32–1.70)	2.25 (1–3.6)	1 (25)
**4b (n = 1)**		2.31	0	0
***p*-value**	–	0.55	0.36	1
**Initial recurrence risk** **(*n*** **= 24)**				
**Low (*n*** **= 21)**	51.5 (18–68)	1.44 (0.32–4.8)	1.25 (0–7)	8 (40)
**Intermediate (*n*** **= 2)**	55.5 (42–69)	0.97 (0.64–1.31)	2.13 (0.66–3.6)	0
**High (*n*** **= 1)**	66	2.31	0	0
***p*-value**	0.33	0.33	0.69	0.68
**Initial response** **(*n*** **= 23)**				
**Excellent (*n*** **= 12)**	58.5 (18–69)	1.4 (0.32–3.09)	0.5 (0–7)	6 (50)
**Indeterminate (*n*** **= 7)**	50 (37–68)	1.34 (0.64–4.4)	0.83 (0–2.3)	3 (42.9)
**Biochemical** **incomplete (*n*** **= 3)**	37 (37–38)	3.37 (1.94–4.8)	3 (0.25–4)	0
**Structural** **Incomplete (*n*** **= 1)**	66	2.31	0	0
***p*-value**	0.40	0.65	0.79	0.42
**Response at the end of follow-up** **(*n*** **= 23)**				
**No evidence of disease (*n*** **= 19)**	50 (18–69)	1.21 (0.32–4.4)	1 (0–7)	7 (36.8)
**Indeterminate (*n*** **= 2)**	65 (62–68)	1.8 (0.98–2.6)	1.8 (0–3.6)	1 (50)
**Biochemical** **incomplete (*n*** **= 1)**	37	1.7	0	0
**Structural** **Incomplete (*n*** **= 1)**	66	2.31	0	0
***p*-value**	0.23	0.30	0.87	0.71

*DTC* differentiated thyroid cancer, *IGF-1* insulin-like growth factor type 1, *ULN* upper limit of normality

When comparing RFU between 23 acromegalics and 92 controls matched for RR (1:4 ratio), no statistically significant differences were found (Table 1). Consistently, an OR of 0.39 (95% CI 0.12–1.26) suggests no impact of the presence of acromegaly on the RFU of DTC (Table 4).

**Table 4:** Comparison of recategorized responses at follow-up between acromegalic and control patients (absolute values and percentages)

Follow-up response	Excellent	Non-excellent	OR	95% CI
**Acromegalics**	19	4	0.395	0.12–1.26
(*n* = 23)	82.7%	17.3%		
**Controls**	60	32		
(*n* = 92)	65.2%	34.8%		

Recategorization of responses at follow-up (American Thyroid Association Guidelines 2015) into excellent (no evidence of disease) and non-excellent (aggregating high risk categories by including indeterminate, biochemical incomplete and structural incomplete responses)

## Discussion

The evolution of thyroid carcinoma in acromegalic patients has not been fully described in the literature. In the presence of acromegaly, a worse evolution could be expected. In the current study, we describe the baseline characteristics of 24 patients with acromegaly and DTC, and explore the follow-up, finding no evidence of a poorer outcome.

Acromegaly is a rare disease with high morbidity and mortality [19]. The lack of biochemical control (GH excess) is associated with increased comorbidities [2]. Over the last few years, there has been a significant improvement in mortality and morbidity due to advances in diagnosis and treatment. Mortality in acromegaly is mainly due to cardiovascular disease (60%), respiratory disease (25%) and malignant disease (15%) [19]. Early diagnosis and an effective therapy are the keys to prevent further comorbidities and ensure a better quality of life and higher survival rates. However, there is still a significant delay in diagnosis [20]. Our patients had a median delay in diagnosis of 3 years (range 0.5–23 years), estimated according to personal history, with baseline IGF-1 at diagnosis of acromegaly of almost 3.0 ULN, and 2.3 ± 1.1 ULN at the diagnosis of DTC.

Different studies have shown a SMR varying from 1.3 to 3.0 [5, 7, 21–23] in acromegaly. Despite a decrease in SMR in modern times, cumulative GH exposure is an important factor in morbidity and mortality [8]. Multimodal treatments together with a careful management of comorbidities have been associated with a decrease in mortality, as shown in a retrospective study of 442 acromegalic patients in Mexico where the SMR was 0.72 (95% CI, 0.41–1.03), cancer being the most common cause of death. The presence of malignant neoplasm was associated with age and basal serum GH levels higher than 10 ng/ml, as well as with the severity of acromegaly [24].

At the moment of diagnosis, 58.3% of our patients had active disease. Both GH and IGF-1 are implicated in cancer promotion through in vitro proliferative effects, with angiogenic and antiapoptotic effects. The incidence of cancer in patients with GH deficiency or Laron Syndrome is null [25]. Renehan et al. showed that women with breast cancer without acromegaly have higher serum GH and IGF-1 levels than women without cancer, and circulating IGF-1 levels within the upper normal range have been associated with a higher risk of breast cancer in premenopausal women [26]. Serum IGF-1 levels within the higher quintile of normality have been associated with a higher risk of prostate cancer, 18 times higher in men older than 60 years of age [27].

Being a rare disease, a large population-based cohort study is needed to determine the real incidence of cancer in acromegaly. Data from the German Acromegaly Registry which included 446 patients (6656 person-years from diagnosis) found that overall cancer incidence was slightly but not significantly lower than in the general population (SIR, 0.75; 95% CI, 0.55 to 1.00; *P* = 0.051) [14]. Another nationwide cohort study including 529 acromegaly cases found cancer incidence rates slightly elevated [15].

Most of our patients had normal thyroid function tests with negative thyroid antibodies. Nevertheless, the thyroid gland changes structurally and functionally in the context of acromegaly. GH and IGF-1 excess induce thyroid proliferation. IGF-1 stimulates the growth of rat thyroid cells in culture and synergizes the stimulation of DNA synthesis induced by TSH and Graves’-IgG [28]. Goiter is described in 25 to 90% of the acromegalics [29], multinodular goiter in 65% [9], and there is a positive correlation between thyroid volume and GH and IGF-1 levels [29]. The risk of developing thyroid nodules increases with the duration of the disease. Almost half of our patients had palpable thyroid nodules. IGF-1 has a proliferative and antiapoptotic effect on thyroid cells due to the presence of its receptor. Different studies have shown an OR of 3.6 (95% CI 1.8–7.4) for nodular thyroid disease compared to controls or patients with other pituitary diseases [30].

Wolinski et al. found thyroid cancer prevalence of 4.3%, higher than in the control group [31]. Tirosh and Shimon [17] also found a higher frequency of thyroid cancer compared with controls (3.2% versus 0.3%), the papillary subtype being more frequent (43/47 tumors), with low mortality rates. In an Argentinian series, thyroid cancer prevalence was of 11% among 34 acromegalics [32]. In summary, different studies estimate thyroid cancer prevalence to be between 1.2 and 11% [32–36]. It should be mentioned that in one of the studies that shows higher prevalence, the authors evaluated nodules suspicious of malignancy smaller than 1 cm in diameter, considering the presence of risk factors for thyroid cancer in the population studied, such as iodine deficiency and the radiation received due to the Chernobyl nuclear accident [34].

Our study is one of the largest published series of DTC in acromegaly, comparing its evolution with that of a control group of DTC patients without acromegaly. All were papillary carcinoma, in coincidence with other studies, with a median time from DTC diagnosis to acromegaly control of 1.25 years. Thyroidectomy was performed in all the patients, and 87.5% received radioiodine ablation.

Compared to the control group, no statistically significant differences were found in gender distribution, history of other cancer, mean age at diagnosis of DTC, histology, tumor size, lymphadenectomy or median time of FU. However, insulin resistance was more frequent in acromegalics, as expected.

In a retrospective evaluation, Mercado et al. described that the most common type of malignancy in acromegaly was thyroid cancer, present in over one-third of patients with cancer. Five out of seven patients with thyroid cancer had classic papillary carcinomas, and two died of anaplastic tumors (one with controlled acromegaly and the other with active disease) [24]. Gullu et al. [37] detected malignancy in 15% of 105 patients with acromegaly, thyroid cancer being the most frequent (4.7%), followed by colon and breast cancer. Cancer was more common in male patients (*P* = 0.046) and high levels of GH increased the risk of cancer development (*P* = 0.046).

In our series, the acromegalics with DTC had a low initial RR, which might be related to an early diagnosis of DTC (anticipated bias), as it occurs in the general population. We did not find any predisposing factors for unfavorable evolution, as we found no statistically significant correlations with stage, RR, initial response or final response at FU. Gul et al., in a retrospective study of 14 acromegalic patients, identified no changes in the disease course and treatment responses of DTC in association with the acromegaly activity, gender, age and disease duration, and all patients were found to be in remission for DTC at the time of investigation [38].

When comparing with the control group, we can conclude that DTC in acromegaly does not have a worse evolution. Additionally, it is worth noting that an OR of 0.39 (95%CI 0.12–1.26) suggests no impact of the presence of acromegaly on the RFU of DTC. Our findings show the same long-term evolution in patients, showing that prognosis is not worse in acromegaly.

The strength of our study is the description of a cohort of 24 patients with an infrequent comorbidity in a rare disease, the largest published series, to the best of our knowledge. However, the main limitation is the combination of few patients with acromegaly and higher risk DTC and the relatively short time of follow-up for thyroid cancer in the acromegaly group.

## Conclusions

Recent studies show a higher rate of thyroid malignancy in acromegaly, which might be due to anticipated diagnosis bias, as seen in the general population. In our series we did not identify any risk factors responsible for worse evolution.

When comparing with the control group, we can conclude that DTC in acromegaly does not have a more aggressive evolution. However, the overall impact of the study is limited by the small number of patients with acromegaly and higher risk DTC. Extension of follow-up time and inclusion of more patients with high risk categories of DTC might provide evidence of the validity of the available data in the long term.

## Supplementary Information

**Additional file 1:** **Supplemental Table 1.** Description of acromegalic patients: Age and IGF-1 at diagnosis, time from DTC diagnosis to acromegaly control, stage, recurrence risk (RR), initial and follow-up (RFU) response of DTC. RR: recurrence risk; RFU: response at the end of follow-up; NA: not available because of brief evolution after surgery which prevents response classification.

## Data Availability

The datasets used and/or analyzed during the current study are available from the corresponding author on reasonable request.

## Abbreviations

DTC: Differentiated thyroid carcinoma
RR: Initial risk of recurrence
RFU: Response at the end of follow-up
MT: Medical treatment
