# Supplementary material for: Acromegaly and thyroid cancer: analysis of evolution in a series of patients
Source: Clin Diabetes Endocrinol. 2020 Nov 17;6:24. doi: 10.1186/s40842-020-00113-4 (PMC7672811; doi:10.1186/s40842-020-00113-4)
Supplement: Supplementary file 1 — Additional file 1: Supplemental Table 1. Description of acromegalic patients: Age and IGF-1 at diagnosis, time from DTC diagnosis to acromegaly control, stage, recurrence risk (RR), initial and follow-up (RFU) response of DTC. RR: recurrence risk; RFU: response at the end of follow-up; NA: not available because of brief evolution after surgery which prevents response classification. [file 40842_2020_113_MOESM1_ESM.docx]

**Supplemental table 1.** Description of acromegalic patients: Age and IGF-1 at diagnosis, time from DTC diagnosis to acromegaly control, stage, recurrence risk (RR), initial and follow-up (RFU) response of DTC

| **Pat.** | **Stage** | **RR** | **Initial**  **response** | **RFU** | **Age at DTC**  **diagnosis (y)** | **Time DTC-**  **acromegaly control**  **(y)** | **IGF-1 at DTC**  **diagnosis (ULN)** |
| --- | --- | --- | --- | --- | --- | --- | --- |
| 1 | I | Low | Biochemical  incomplete | Biochemical  incomplete | 37 | 0.25 | 4.8 |
| 2 | II | Low | Excellent | No evidence of  disease | 64 | 3 | 1.7 |
| 3 | I | Low | Excellent | No evidence of  disease | 58 | - | 0.4 |
| 4 | I | Low | Indeterminate | No evidence of  disease | 43 | 1 | 0.9 |
| 5 | I | Low | Biochemical  incomplete | No evidence of  disease | 37 | 3 | - |
| 6 | II | Low | Indeterminate | No evidence of  disease | 58 | 1 | 1.6 |
| 7 | I | Low | Indeterminate | No evidence of  disease | 50 | - | 4.4 |
| 8 | I | Low | Excellent | No evidence of  disease | 35 | - | 1.1 |
| 9 | I | Low | Excellent | No evidence of  disease | 62 | - | 0.7 |
| 10 | I | Low | Excellent | No evidence of  disease | 36 | 0.5 | 1.1 |
| 11 | I | Low | Excellent | No evidence of  disease | 18 | 7 | 3.1 |
| 12 | II | Intermediate | Excellent | No evidence of  disease | 69 | 3.6 | 1.3 |
| 13 | I | Low | Indeterminate | No evidence of  disease | 37 | 0.5 | 1.3 |
| 14 | I | Low | Indeterminate | No evidence of  disease | 68 | - | 1 |
| 15 | I | Low | Biochemical  incomplete | Indeterminate | 38 | 4 | 1.9 |
| 16 | IVb | High | Structural  incomplete | Structural  incomplete | 66 | - | 2.3 |
| 17 | I | Intermediate | Indeterminate | No evidence of  disease | 42 | 0.7 | 0.6 |
| 18 | I | Low | Indeterminate | No evidence of  disease | 58 | 2.3 | 1.9 |
| 19 | I | Low | Excellent | No evidence of  disease | 37 | - | 0.3 |
| 20 | II | Low | Excellent | No evidence of  disease | 67 | 1.5 | 1.4 |
| 21 | I | Low | Excellent | Indeterminate | 62 | 3.6 | 2.6 |
| 22 | I | Low | NA | NA | 43 | - | 1.3 |
| 23 | I | Low | Excellent | No evidence of  disease | 59 | - | 1 |
| 24 | I | Low | Excellent | No evidence of  disease | 53 | - | 2.2 |

RR: recurrence risk; RFU: response at the end of follow-up; NA: not available because of brief evolution after surgery which prevents

response classification
